# Stable Small Animal Ventilation for Dynamic Lung Imaging to Support Computational Fluid Dynamics Models

**DOI:** 10.1371/journal.pone.0027577

**Published:** 2011-11-08

**Authors:** Richard E. Jacob, Wayne J. Lamm

**Affiliations:** 1 Biological Monitoring and Modeling, Pacific Northwest National Laboratory, Richland, Washington, United States of America; 2 University of Washington School of Medicine, Seattle, Washington, United States of America; University of Giessen Lung Center, Germany

## Abstract

Pulmonary computational fluid dynamics models require that three-dimensional images be acquired over multiple points in the dynamic breathing cycle without breath holds or changes in ventilatory mechanics. With small animals, these requirements can result in long imaging times (∼90 minutes), over which lung mechanics, such as compliance, may gradually change if not carefully monitored and controlled. These changes, caused by derecruitment of parenchymal tissue, are manifested as an upward drift in peak inspiratory pressure (PIP) or by changes in the pressure waveform and/or lung volume over the course of the experiment. We demonstrate highly repeatable mechanical ventilation in anesthetized rats over a long duration for dynamic lung x-ray computed tomography (CT) imaging. We describe significant updates to a basic commercial ventilator that was acquired for these experiments. Key to achieving consistent results was the implementation of periodic deep breaths, or sighs, of extended duration to maintain lung recruitment. In addition, continuous monitoring of breath-to-breath pressure and volume waveforms and long-term trends in PIP and flow provide diagnostics of changes in breathing mechanics.

## Introduction

Several reports have been published about mechanical ventilation of small animals for pulmonary imaging, with the primary focus often on optimizing resolution [1] or delivering special gas mixtures [2], [3]. Static or quasi-static imaging, in which data are acquired during extended or brief breath holds [4], [5], is typical in these approaches, resulting in high quality images of lung structure in health and disease. As an alternative to mechanical ventilation, respiratory gating (prospective or retrospective) during free-breathing can be used [6], [7]; however, it is shown that respiratory dynamics are variable breath-to-breath, even under well-controlled conditions [8], [9]. Although basic principles of small animal ventilation are well established, little attention has been placed on ensuring or demonstrating stability of lung mechanical properties throughout long imaging experiments.

Current work in our group is focused on generation of computational fluid dynamics (CFD) models of lung mechanics, airflow, and particle deposition [10], [11], [12]. These models not only demand high-resolution images of conducting airway geometry (which are typically acquired from images of airway casts [13]), but also require three-dimensional (3D) images of the intact breathing lung, acquired during inhalation and exhalation with adequate temporal resolution (≤100 ms), to define the hysteretic breathing cycle. Images are then used to calculate 4D (three spatial dimensions in time) tissue strain and compliance maps, which can be applied to determine airflow boundary conditions and mechanical inputs, such as compliance and strain, for the CFD models. Requirements for data collection, therefore, demand that images be acquired dynamically (i.e. without breath holds) and that the lung mechanics are consistent throughout the imaging experiment, which typically run about 90 minutes or more. In addition, sufficient contrast is necessary to discern the major airways that feed the five lobes of the rat lung so that lobar-specific parameters can be calculated. While the CFD models rely on feature recognition algorithms, of secondary concern is overall image resolution, although undue blurring from lung motion can confound image analysis.

We have demonstrated a method of ventilating anesthetized rats for long x-ray computed tomography (CT) imaging sessions that results in stable, consistent tidal pressure and volume waveforms and very little long-term drift in peak inspiratory pressure (PIP). Using a customized commercial ventilator, we acquired 11 dynamic CT images with 80–100 ms temporal resolution while striking a balance between imaging time and image quality. Longer imaging times are necessary for higher quality images, which then benefit the CFD models; however, they increase the potential for physiological variability of breathing mechanics, which can confound computational analysis. This approach would also be useful in magnetic resonance imaging (MRI) of lung.

## Materials and Methods

### Ethics Statement

All animal work followed protocols submitted to and approved by the Institutional Animal Care and Use Committee at Pacific Northwest National Laboratory.

### Ventilator

A commercial ventilator from CWE Inc. (Model 830/AP; Ardmore, PA) was acquired and customized for this project. The ventilator was interfaced with a PC using a USB-6008 card and LabVIEW software (National Instruments, Austin, TX). Features of the ventilator and graphical user interface (GUI) included: two different breathing cycles (labeled A and B) with independent controls of breathing rate but a universal control of percent inspiration; a 0–5 V gating signal with adjustable position and duration for synchronization with external hardware, such as an imaging system; a pressure-limited sigh (a deep breath of extended duration to promote alveolar recruitment) that can be implemented manually or automatically after a fixed number of breaths (the gate is muted on a sigh breath); an over-pressure limit to avoid over-distending the lungs; manual inhale, exhale, and breath hold controls; the ability to switch to a manually adjustable, low-pressure external regulator intended for instituting long breath holds at a constant user-determined pressure (for use during breath hold imaging); and a running plot of the measured tracheal pressure, gate position, and inhale/exhale valve status. We note that the pressure-limited sigh was of undetermined duration, depending on factors such as the air flow rate and the sigh pressure limit, but these sighs were typically ∼2 seconds duration.

**Figure 1:**
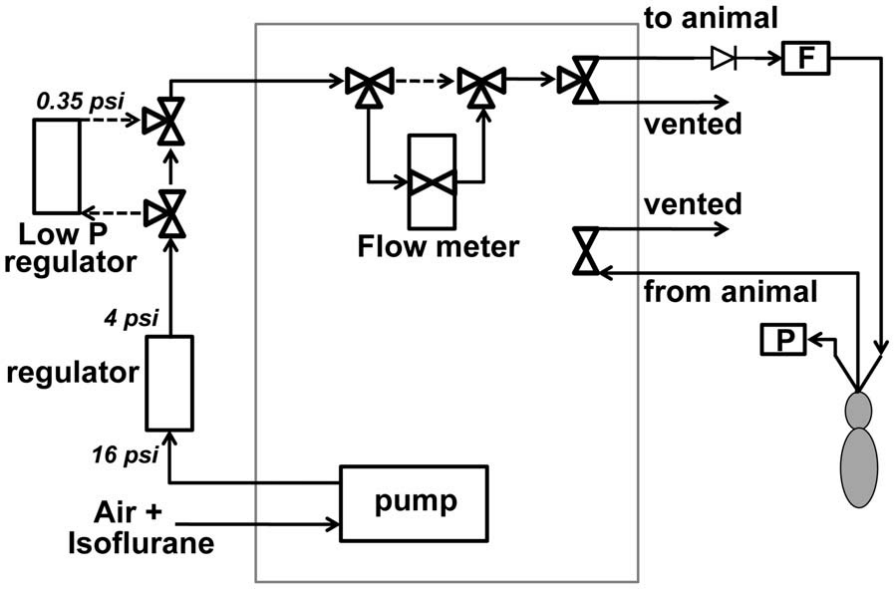
Schematic diagram of the airflow paths of the ventilator (the box indicates the extent of the ventilator chassis). Two- or three-way solenoid valves control the airflow. “F” indicates the flow meter, or pneumotachograph, and “P” indicates the pressure transducer. A check valve, immediately upstream from the pneumotach, assures unidirectional flow. Vented air is passed through filters to remove isoflurane. The dashed lines indicate the flow path during sigh breaths, in which the low-pressure regulator is employed and the needle valve in the flow meter is bypassed to facilitate more rapid inflation.

Several significant changes and upgrades were made to the ventilator hardware. A pneumotachograph and check valve were added to the inspiration line to measure flow rate and to assure unidirectional flow, respectively (see Figure 1). A regulator was added to reduce the line pressure (from a diaphragm pump) from 16 psi to about 4 psi to avoid flow and pressure spikes. The pressure transducer, which was originally connected directly to the circuit board inside the ventilator chassis, was removed and reconnected via a shielded cable in order to be placed nearer the animal. This shortened the distance from the trachea tube to the transducer from approximately two meters down to about 20 cm, thus providing a more precise measure of tracheal pressure.

A considerable number of changes were made to the ventilator control software and GUI. In particular, the LabVIEW source code (provided by CWE Inc.) was customized. We added code to integrate the flow rate signal from the pneumotach to calculate total inhale volume during each breathing cycle. Importantly, the flow rate signal is automatically zeroed, or tared, at the beginning of each inhale cycle to minimize accumulated errors in the volume calculation that are caused by slight drift in the pressure sensor reading. The user can calibrate the pneumotach using the flow meter on the ventilator chassis and calibrate the calculated flow volume using syringes of known volumes. This is done before each use, although we have found that the calibrations change very little over time. The running plot on the GUI was then modified to include the flow rate. In addition, a numerical display shows the PIP and total inhale volume updated with each breathing cycle. A second plot was added to show the long-term trend in PIP and volume; this plot is updated once per breath at the end of each cycle and shows data from the previous 500 breaths. All data are saved to spreadsheet files for later analysis, if necessary. In addition, all user-defined ventilator parameters (breathing rate, inspiration time, etc.) are saved to a file.

A critical upgrade was to the method of producing periodic sigh breaths, used as alveolar recruitment maneuvers. As noted above, the original configuration provided a brief sigh up to a fixed pressure, and the sigh would end as soon as that pressure was reached. We found that this was ineffective in preventing upward drift of the PIP over several minutes, presumably caused by decreasing lung compliance from ongoing atelectasis in lung that was not adequately recruited [14], [15]. We reconfigured the ventilator to generate a sigh of user-defined duration and included a pressure plateau at peak inspiration. Importantly, flow is not stopped during the sigh plateau, but is regulated at a fixed pressure in order to maximize lung recruitment while avoiding damage from over extension. This was done by exploiting the external low-pressure regulator that was originally intended for breath hold imaging. Now, during a sigh breath, the ventilator automatically switches for a single breath from the A cycle, used for regular breathing, to the B cycle, which can be set to a longer duration and a higher percent inspiration. Simultaneously, the flow path switches to the low-pressure regulator and bypasses the flow meter in order to maximize the inspiratory flow rate (see Figure 1). The low-pressure regulator is used to set and maintain the peak sigh pressure, and it can be easily adjusted by the user with ∼0.1 mH_2_O precision. A numerical display on the ventilator GUI shows the peak pressure and volume of the most recent sigh, while all volumes and pressures are recorded to a data file. In addition, a gating mute was added to prevent a gate signal from being sent for a user-specified number of breaths after the sigh. Since PIP can decrease immediately following a sigh breath [15], the mute allows time for the lung to relax back closer to its normal steady-state condition prior to resuming image collection.

A trial and error approach was used to find optimal ventilator settings (such as breathing rate, sigh duration, peak inspiratory pressure, ratio of inhale to exhale time, sigh frequency, and inflation flow rate) to be used for long-duration imaging, particularly while modifications were being made to the ventilator. Approximately 20 rats were used in testing and refining of ventilator performance in conjunction with our imaging protocol. Final ventilation parameters were: 60 breaths per min (400 ms inspiration, 600 ms expiration), ∼8 cmH_2_O PIP, 4 sec sigh duration (2800 ms inspiration, 1200 ms expiration), ∼25 cmH_2_O peak sigh pressure, a sigh frequency of once every 100 breaths, and a 5 breath post-sigh gate mute. Sigh breaths were instituted with a relatively high frequency in order to maintain steady ventilatory mechanics [15]. We note that the upward PIP drift was better controlled in some rats with more frequent sighs, as often as once every 75 breaths; this rate could be adjusted at any time during the experiment. The over-pressure limit was set to 30 cmH_2_O to prevent any accidental over inflation, which can occur if the sigh-limiting regulator is initially set improperly. The inspiratory volume was animal dependent, but was typically within the range of 8±2 mL/kg.

### Animal Preparation

In this paper we discuss results from male Sprague-Dawley rats (260–425 g), although early development work was also done with male Lewis rats of a similar weight range. Rats were anesthetized with 3–4% isoflurane in oxygen, then intubated with a 14 gauge catheter tube, laid supine in the imager's animal tray, and connected to the ventilator. The ventilator delivered a mixture of 30% O_2_ and 70% N_2_. To maintain compliance with the ventilator, the anesthesia level was kept somewhat high, at 3–4%. We have observed anesthetized rats begin to struggle against the ventilator even after many minutes of compliant breathing if the anesthesia level is not maintained at a sufficiently high level (Sprague-Dawley rats seemed more prone to this than the Lewis rats). Rats usually required 5–7 minutes to become totally compliant with the ventilator before imaging could begin. Body temperature was maintained by a custom-made pad that circulated warm water beneath the animal and provided a blanket-like cover above to protect from drafts in the imaging tube caused by cooling fans in the scanner. A dual-temperature warm water circulator (T/Pump, Gaymar Industries, Orchard Park, NY) was customized to provide a wider range of temperature control. Body temperature, typically maintained at 37±1°C, and pulse were monitored (SAII, Stony Brook, NY).

### CT Imaging

A commercial CT scanner (eXplore CT120, GE HealthCare, Waukesha, WI) was used for all imaging. The entire lungs of the rats at full inspiration were easily contained within the field of view. Imaging settings were: 100 kVp, 50 mA, 16 ms exposure time, 360 projections with 1° increments, and 2×2 binning. Images were acquired at 50 µm resolution and reconstructed to 150 µm isotropic resolution to improve contrast-to-noise.

During imaging, the ventilator sent a single gate at the beginning of each breathing cycle, and the CT scanner acquired a single projection at a given gantry angle per gate event. To acquire the 11 images over the breathing cycle, the CT scanner was programmed with 11 trigger delays. Each trigger delay represented one image at a specified time point in the breathing cycle. Thus, 11 breaths were required for the 11 exposures taken at a single gantry angle. After the sequence of trigger delays was completed, the gantry incremented to the next angular position and the trigger delay sequence repeated. Gantry motion required ∼1.5 sec, and any gates received during that time were ignored. This approach of only one projection per breath was necessary due to CT scanner firmware limitations allowing only one exposure per gating event. Optimally, imaging time could be reduced considerably if multiple exposures per gating event were possible. For a full 360° scan and 11 images, total imaging time was about 90 minutes. Trigger delays were typically set at: 0, 80, 160, 240, 320, 400, 500, 600, 700, 800, and 900 ms from the beginning of each breathing cycle. We measured the delay between when the scanner receives a gate signal to when x-rays fire to be <250 µs.

## Results

**Figure 2:**
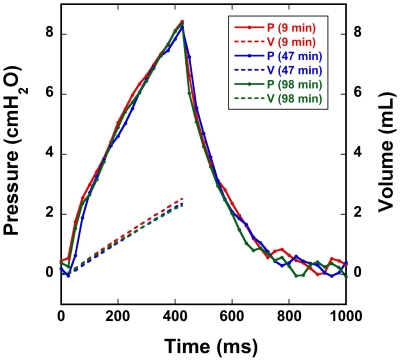
Three pressure waveforms and corresponding inspiratory flow volumes, taken from different points during an imaging experiment with a 306 g rat are shown. The “noise” at the tail end of the pressure traces is due to the beating heart. The strong similarity between waveforms demonstrates the consistency of lung mechanical properties over the course of the experiment.

To demonstrate stability of the pulmonary mechanics over time, Figure 2 shows three pressure waveforms, with accompanying inhale volumes, for three breaths at different points during an imaging experiment with a 306g rat. The breaths were taken from near the beginning (9 min), middle (47 min), and end of imaging (98 min). For consistency, each waveform was taken from the same relative position within a sigh-sigh interval, but no other criteria were used in their selection. This figure shows virtually no changes in the shape of the pressure waveform or drift in PIP over the duration of the experiment. We note that the pulse pressure generated from the heart picked up by the tracheal pressure transducer can cause “noise” fluctuations of approximately 0.1–0.2 cmH_2_O.

**Figure 3:**
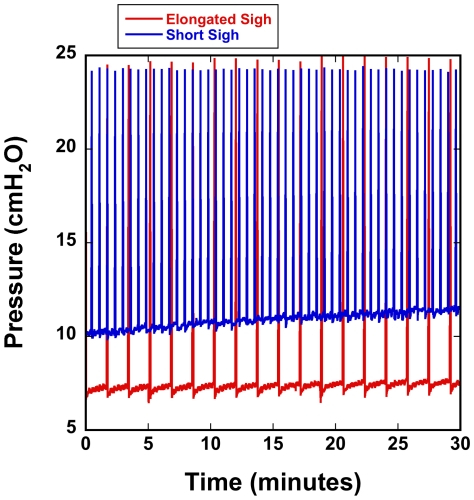
Peak inspiratory pressure (PIP) from two rats over 30 minutes of ventilation. One rat was given frequent but brief sighs, every 30 breaths, and the other was given elongated sighs every 100 breaths. This figure shows that elongated sigh breaths were more effective at mitigating long-term drift in PIP.

As noted above, we found that an elongated sigh with a breath hold at peak pressure was more effective at maintaining consistent ventilation mechanics than a brief sigh that terminated after reaching a set pressure. Figure 3 shows the trend in PIP and peak volume of two rats. One rat was given elongated sighs (24–25 cmH_2_O) every 100 breaths, and the other brief but more frequent sighs (24 cmH_2_O, every 30 breaths). The increased frequency was originally an effort to combat the PIP drift. When using the brief sigh, we routinely saw long-term PIP drifts of >2 cmH_2_O/hour, whereas with elongated sighs we commonly observe long-term upward drift in the PIP of about <0.3 cmH_2_O/hour. When no sigh was used, we observed drift in PIP of approximately 6 cmH_2_O/hour.

**Figure 4:**
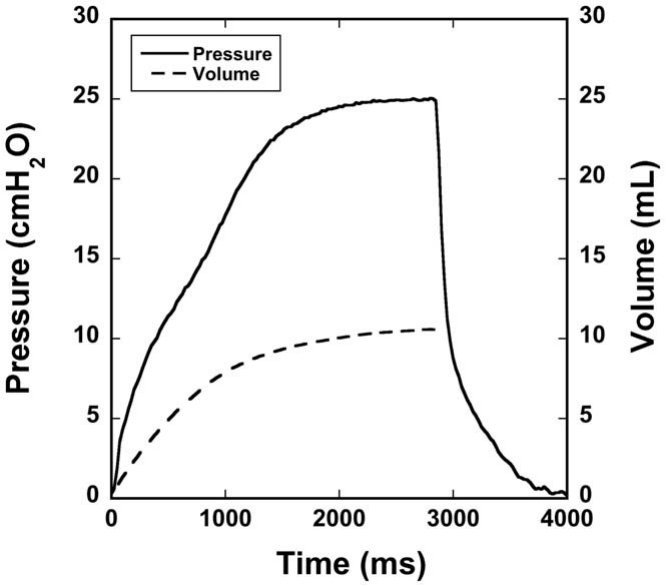
Pressure and volume waveforms of a typical 4 sec sigh breath. The inhale duration is 2800 ms, and peak pressure is limited by the low-pressure regulator.

An example of an elongated sigh breath is shown in Figure 4. The pressure and inhale volume curves from a typical 4 sec sigh breath up to about 25 cmH_2_O are shown. Inhale time was 2.8 sec, and exhale time was 1.2 sec.

**Figure 5:**
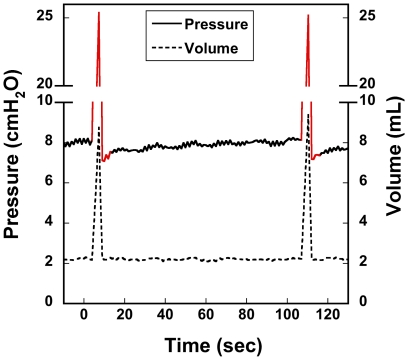
A sample segment of the long-term trend data of peak pressure and inhale volume. An arbitrary t = 0 starting point was chosen on the x-axis to illustrate the timing of the sigh-sigh interval. The red sections show the duration of the gate muting, when no imaging took place during the sigh and for five breaths following. The modulations on the pressure line are due to constructive/destructive interference with the heartbeat.

Figure 5 shows typical PIP and peak volume trend data of a 100-breath sigh-sigh interval. Gate-muted breaths are highlighted in red. In between sigh breaths, the PIP can range as much as ∼0.5 cmH_2_O; this does not include gate-muted breaths. We note that constructive/destructive interference of the pulse pressure with the tracheal pressure adds to apparent fluctuations in measured pressure.

**Figure 6:**
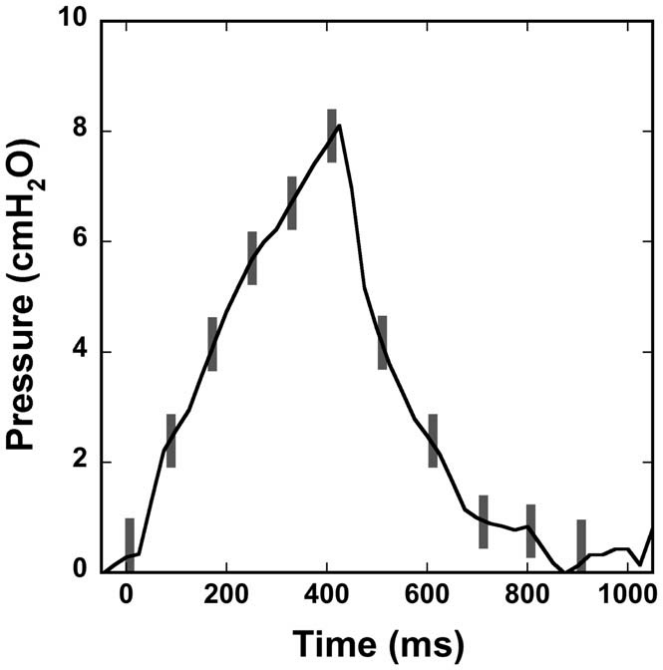
The pressure waveform of a typical single breath with the trigger positions (grey bars) superimposed. The width of the bars represents the 16 ms duration of the x-ray exposure.

Figure 6 illustrates a single breath pressure waveform with the 11 x-ray exposure positions superimposed as vertical bars. The width of the bars represents the duration of the exposures (16 ms). The change in tracheal pressure during a typical exposure is approximately 0.4–0.5 cmH_2_O, and this corresponds to a volume change of about 0.10–0.12 mL, or roughly 5% of the tidal volume.

**Figure 7:**
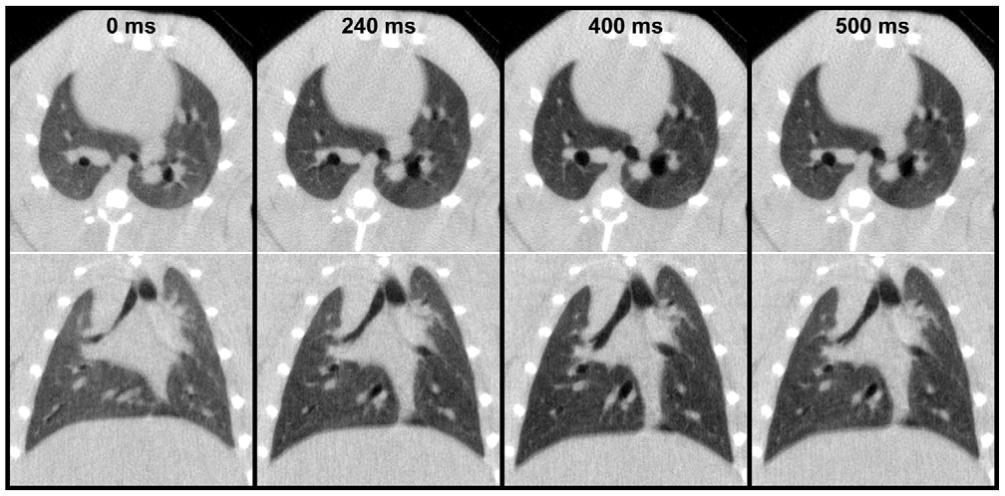
Examples of transverse (top) and coronal (bottom) images of a 359g rat. The images were taken from four different points (out of 11 total) in the breathing cycle. Reconstructed resolution is 150 µm, isotropic.

Figure 7 shows an example of four transverse and four coronal CT images from a 359 g rat at four representative time points (0, 240, 400, and 500 ms). These images show time points near full expiration (0 ms), peak inspiration (400 ms), and points in between during inhale (240 ms) and exhale (500 ms). These images demonstrate the degree of motional blurring that occurs during the 16 ms exposure time for the inhale/exhale images, particularly where the pressure is changing rapidly (240 and 500 ms). As expected, we notice the greatest amount of blurring at the base of the lung, where the motion is most rapid, and immediately around the heart. We note that the pressures at 240 ms and 500 ms are considerably different (see Figure 6) even though the lung is very similar in size in both images. This is attributed to breathing cycle hysteresis.

## Discussion

For CFD models of pulmonary mechanics and airflow, experimental imaging data are needed to provide parameters such as lung structure and geometry, lobar compliance, and tissue strain. To assure realistic models, it is important to maintain consistent, repeatable breathing mechanics throughout the entire duration of the imaging. When high temporal resolution is required, this translates to thousands of breathing cycles. For example, our most intensive imaging experiment requires an anesthetized rat to be ventilated for about 6000 cycles with virtually no changes in breathing mechanics occurring. We have demonstrated consistency and repeatability in breathing mechanics for the duration of such imaging experiments. Key to these efforts was the implementation of the periodic extended sigh for lung recruitment. It has been demonstrated that periodic deep inflations can reverse alveolar derecruitment without adversely affecting the lung [16]. We observed that quick sighs that terminated upon reaching a pressure limit were ineffective at preventing long term pressure drift (see Figure 3). We found that a 25 cmH_2_O sigh of a few seconds duration repeated every 75–100 breaths was adequate for maintaining consistent pressure waveforms over time and minimizing PIP drift. This pressure is well below the limits where ventilator induced lung damage is shown to occur [17]. Also important to the ventilation consistency was maintaining adequately high level of anesthesia and avoiding ventilating with pure oxygen.

An inherent problem with lung imaging is motion, since the lung and heart are dynamic organs. Others have shown that, even under the most ideal conditions, optimal resolution of live animal lung imaging is about 50–100 µm [18]. Although motional blurring cannot be avoided, it can be minimized. This becomes a particular challenge during extended imaging experiments, as gradual changes in breathing mechanics can compound the already inherent blurring and induce motion artifacts. Therefore, controlling or minimizing these changes is important to optimizing image quality. Also key in minimizing blurring during dynamic imaging is a short data acquisition time. Our CT system was limited to a minimum 16 ms exposure; however, we observed no appreciable differences in image quality for images acquired during rapid inhalation/exhalation versus images acquired at peak inhalation or full exhalation (see Figure 7). We note that image acquisition was avoided during the steepest part of the pressure curve: the first ∼100 ms following the onset of exhalation (see Figure 6).

An alternative to dynamic imaging is the quasi-static approach of imaging during breath holds at multiple inflation levels, primarily used when very high-resolution images are desired. This may require either extended breath holds, which may not be feasible for animal well-being, or short breath holds during each breathing cycle, which can considerably lengthen the experiment time. It has been shown that the lung can relax significantly even during short breath holds, so some degree of motion artifacts may still be unavoidable [9]. Additionally, these quasi-static approaches do not represent the dynamic lung required for CFD models, as lung mechanical properties are frequency dependent [16].

These imaging experiments have not been repeated on the same animal, as lung casts are made immediately after imaging to provide detailed CFD airway geometries. Therefore, any subtle, acute damage to the lung tissue due to the long imaging session is of little concern. However, in longitudinal experiments, or experiments where repeated imaging is necessary, the issue of potential ventilator induced lung damage is important. We note that others have demonstrated minimal histological changes in rats ventilated for up to 5 hours with similar tidal volumes and peak pressures as used herein [17], [19], [20].

In conclusion, we have demonstrated that small animal ventilation can be consistent and stable for long durations for the purpose of dynamic pulmonary imaging. We found that the primary factor for accomplishing this in anesthetized rats was periodic, deep, extended sigh breaths to retain alveolar recruitment.
